# A Rare Case of Colon Adenocarcinoma Metastases to the Trachea: A Case Report

**DOI:** 10.7759/cureus.106857

**Published:** 2026-04-11

**Authors:** Alexandria S Page, Natalie M Wall, Miles R Neumann

**Affiliations:** 1 Department of Osteopathic Surgical Specialties, College of Osteopathic Medicine, Michigan State University, East Lansing, USA; 2 Department of Otolaryngology - Head and Neck Surgery, Henry Ford Warren Hospital, Madison Heights, USA; 3 Department of Otolaryngology/Facial Plastic Surgery, Ear Nose and Throat Consultants, Southfield, USA

**Keywords:** adenocarcinoma of the colon, airway metastasis, globus sensation, tracheal metastases, tracheal tumor

## Abstract

Primary tracheal malignancies are rare, and nonpulmonary metastasis to the trachea is exceedingly rare. Computed tomography (CT) imaging may not detect small or submucosal lesions, especially if performed without contrast. We present a rare case of metastasis of colorectal adenocarcinoma to the trachea and left mainstem bronchus 10 years after the initial cancer diagnosis.

A 75-year-old man presented with a one-month history of globus sensation, a perception of an object ball-valving in his chest, and the ability to produce an audible noise with forced exhalation. He had a history of stage IV (T3N0) colon cancer that was diagnosed 10 years prior. He subsequently underwent a right colectomy and was disease-free for five years, at which time he had an isolated pulmonary metastasis. A left upper lobectomy was performed. A flexible fiberoptic laryngoscopy was performed in the office, and a small, nonraised epiglottic lesion was observed. The reported audible noise upon exhalation was also appreciated. CT chest imaging revealed an intraluminal tracheal lesion. The patient was taken to the operating room for a microsuspension direct laryngoscopy and bronchoscopy with biopsies. Three tracheal and left mainstem bronchus lesions were removed and final pathology demonstrated moderately differentiated adenocarcinoma.

Although extraordinarily rare, tracheal malignancies and metastases should remain in the differential during the workup of a patient with globus sensation. Additionally, further workup with imaging and direct visualization in the operating room is of the utmost importance in patients with a history of malignancy.

## Introduction

Colorectal cancer is the third most common cancer worldwide and is a leading cause of cancer-related mortality [1]. Metastasis of colorectal adenocarcinoma to the upper airway is exceedingly rare, with only a handful of cases reported in the literature, and is thought to occur primarily via hematogenous dissemination [2]. Airway metastases may present diagnostic challenges due to their unlikely location and nonspecific symptoms such as cough, dyspnea, wheezing, and hemoptysis, and standard imaging modalities, including computed tomography (CT), may fail to detect such lesions [3]. This report describes a case of colorectal adenocarcinoma metastasis to the trachea and left mainstem bronchus that was initially only partially detected by CT and ultimately identified during endoscopic evaluation. This case highlights the diagnostic value of endoscopy in patients with persistent upper airway symptoms and a history of malignancy.

## Case presentation

A 75-year-old man with a past medical history of hypertension, type 2 diabetes mellitus, and stage IV (T3N0) colon cancer, staged according to the American Joint Committee on Cancer TNM classification [4], underwent a right colectomy and remained disease-free for five years. After five years, he underwent a left upper lobe lobectomy for an isolated pulmonary metastasis and displayed no clinical evidence of recurrence. Five years after the lobectomy of his left upper lung, he presented to an otolaryngologist with a persistent globus sensation and throat clearing, the sensation of a ball-valving object in his trachea, and an associated noise upon rapid exhalation. These symptoms, particularly the sensation of airway obstruction or "ball-valving," may represent features concerning for an underlying airway lesion. All reported symptoms were present for one month. Given the patient’s oncologic history and persistent concerning symptoms, flexible fiberoptic laryngoscopy evaluation was performed, which revealed a small, nonraised lesion on the laryngeal surface of the epiglottis, in addition to laryngeal candidiasis. During laryngoscopy, the patient was able to elicit the ball-valve sensation and audible noise with rapid exhalation, although no additional glottic or supraglottic etiologies were appreciated. The head and neck exam was otherwise negative, including no appreciated lymphadenopathy. The patient was referred to a pulmonologist for further investigation of symptoms, and a CT of the neck/chest was ordered. He was treated with fluconazole 100 mg daily for one week and started on famotidine 40 mg nightly. He returned after two weeks, reporting no improvement in his symptoms and a negative workup by the pulmonologist.

**Figure 1:**
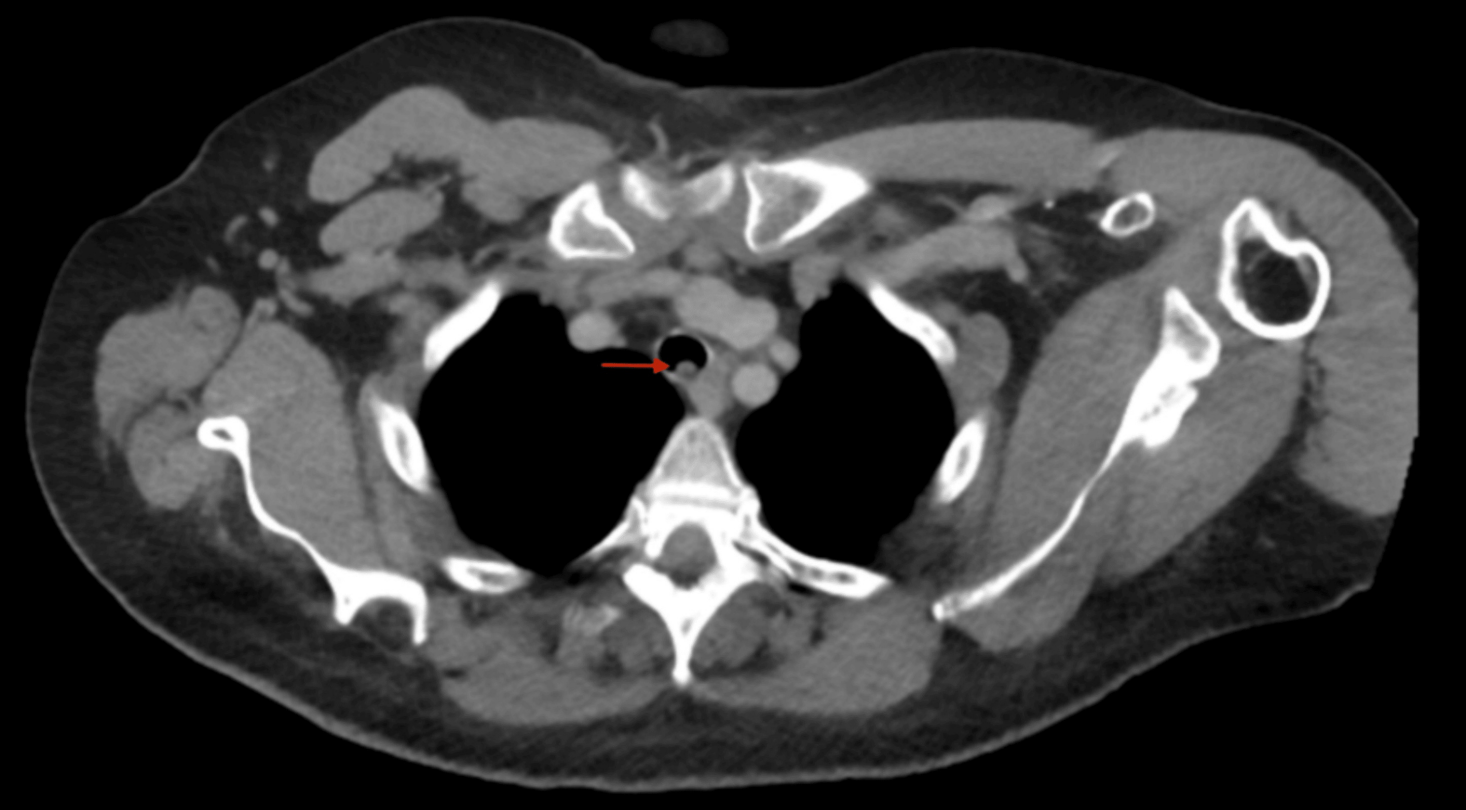
Axial view of the patient's noncontrast CT chest imaging. A metastatic tracheal lesion (red arrow) is demonstrated. The left mainstem bronchus lesion was not detected CT: computed tomography

**Figure 2:**
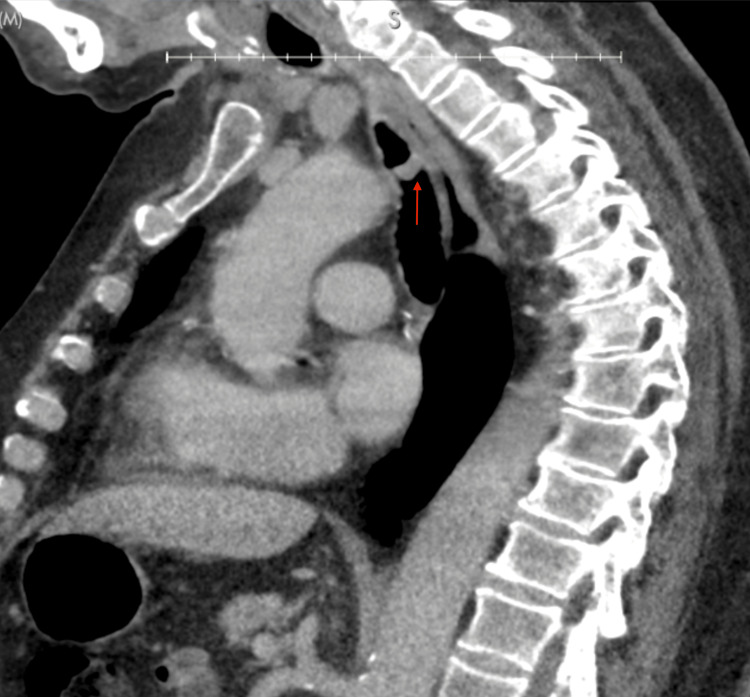
Sagittal view of the patient's noncontrast CT chest imaging. A metastatic tracheal lesion (red arrow) is demonstrated. The left mainstem bronchus lesion was not detected CT: computed tomography

**Figure 3:**
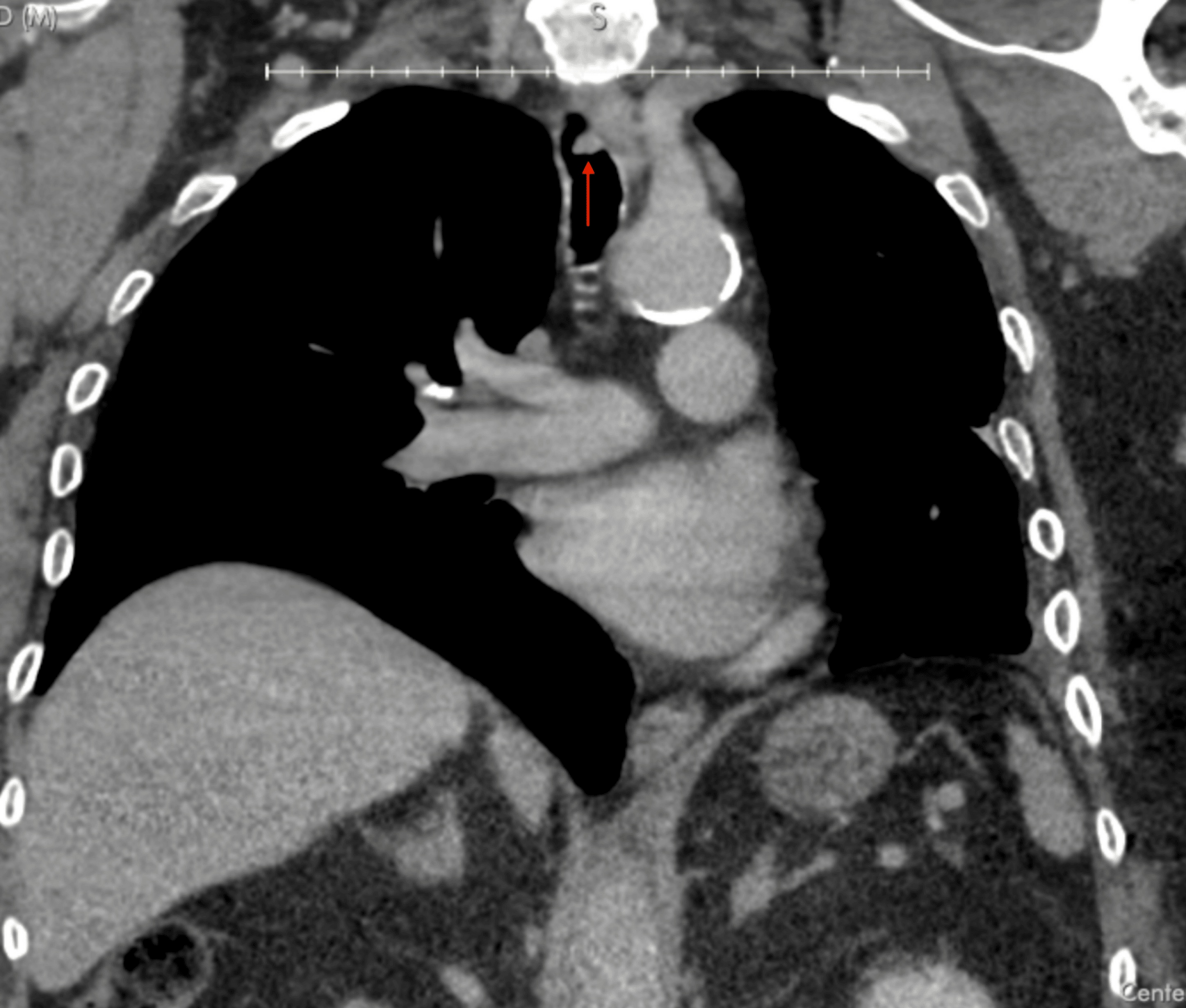
Coronal view of the patient's noncontrast CT chest imaging. A metastatic tracheal lesion (red arrow) is demonstrated. The left mainstem bronchus lesion was not detected CT: computed tomography

**Figure 4:**
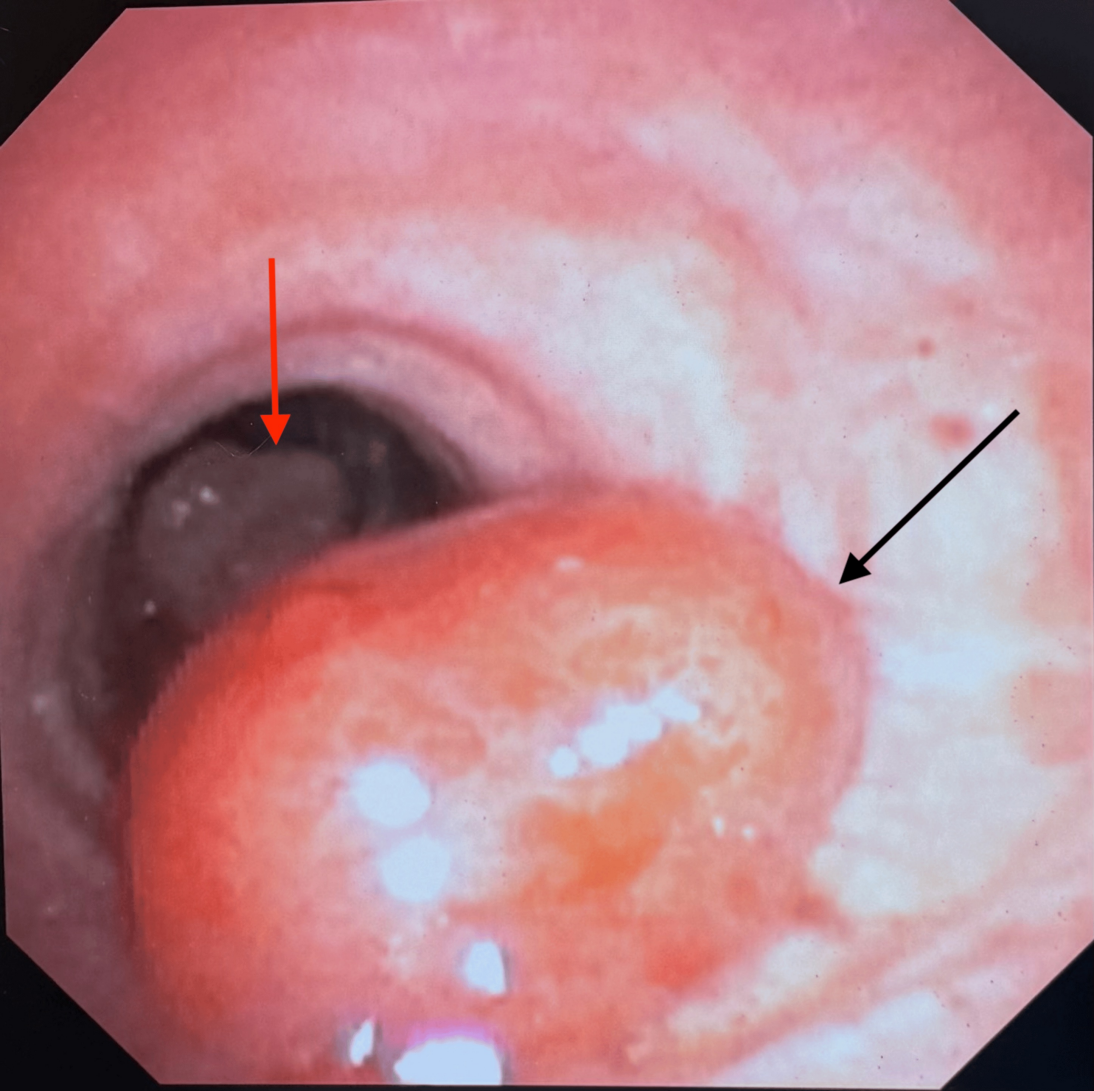
Bronchoscopy revealing a posteriorly based mid-tracheal lesion (black arrow). A distal tracheal lesion was also revealed and may be seen beyond the proximal lesion (red arrow)

**Figure 5:**
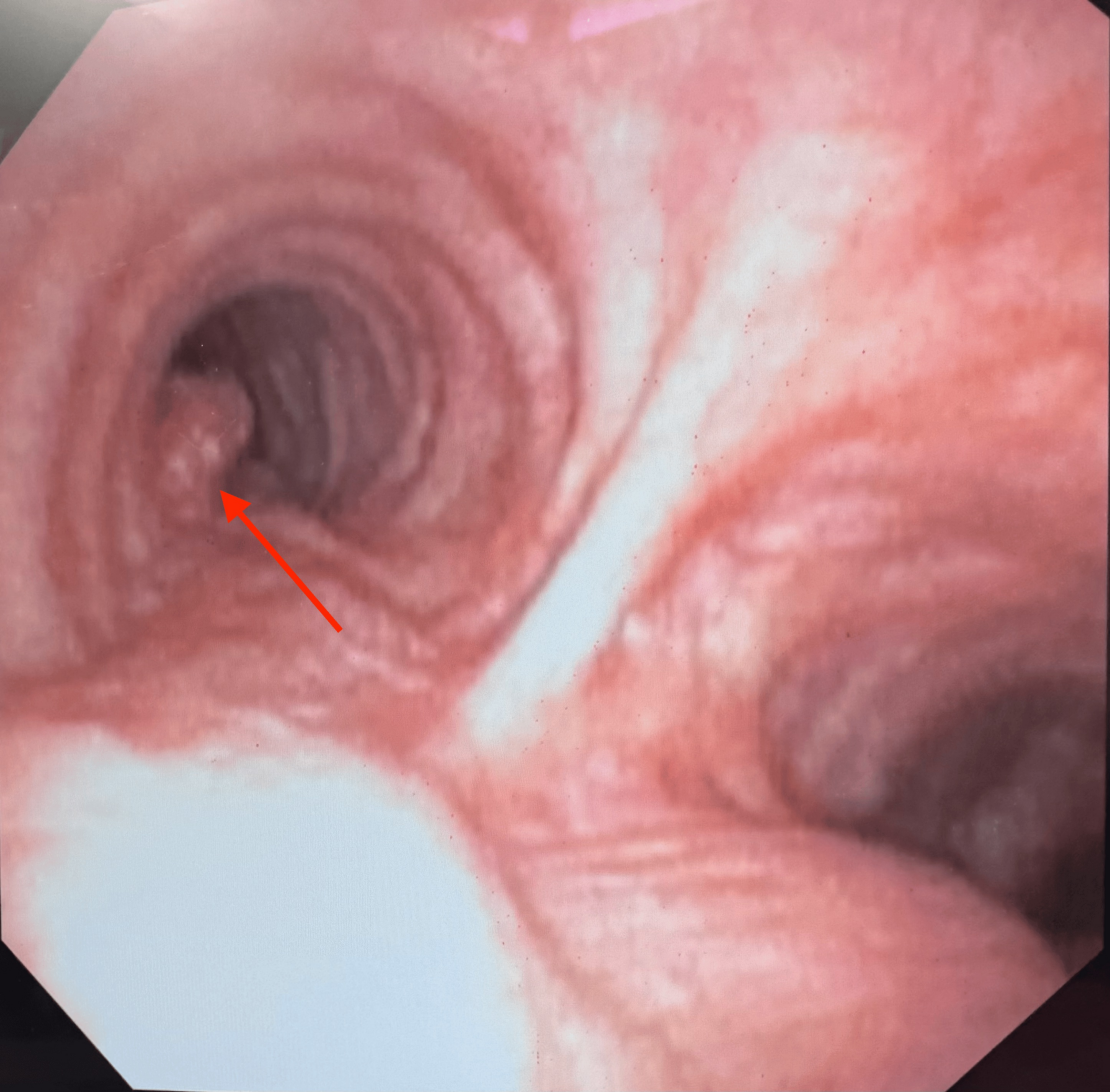
A bronchoscopic view of the bilateral mainstem bronchi. A posterolaterally based lesion may be appreciated in the left mainstem bronchus (red arrow)

The CT of the neck/chest (Figures 1-3) demonstrated two nonobstructive masses in the trachea. He subsequently underwent microsuspension direct laryngoscopy and bronchoscopy with biopsies of the epiglottis and trachea. Direct laryngoscopy revealed a small erythematous lesion on the laryngeal surface of the left epiglottis near its tip. Bronchoscopy demonstrated three lesions (Figures 4, 5): a smooth-edged, posteriorly based pedunculated lesion in the mid-trachea, and two papillomatous-appearing lesions involving the distal trachea and left mainstem bronchus. The masses were excised at their bases using a flexible bronchoscopy and biopsy forceps under apneic technique. Histopathologic analysis of the left epiglottal lesion revealed squamous mucosa with reactive features, negative for dysplasia. The tracheal and left mainstem bronchus lesions revealed moderately differentiated adenocarcinoma, consistent with metastasis from the patient’s known primary colon cancer. The patient underwent a positron-emission tomography (PET)/CT approximately one month after the procedure, which was negative for malignancy or metastatic disease. Carcinoembryonic antigen, a protein used as a biomarker for colorectal cancer, was also negative. The patient remains under the close surveillance of his oncologist. He will undergo a repeat PET/CT in three months, and is considering induction chemotherapy for future treatment.

## Discussion

Colorectal cancers most frequently metastasize to the liver, followed by the lungs, bone, and brain [1]. The overall prevalence of metastasis from nonpulmonary malignancies to the tracheobronchial tree is only about 2%, with colorectal primaries representing an exceedingly small fraction of these cases. Metastatic involvement of the trachea from colorectal adenocarcinoma may occur as a result of hematogenous spread and is rare enough that the incidence cannot currently be accurately determined [2].

CT is the imaging modality of choice for the trachea and bronchi because it provides a clear picture of airway anatomy and can specifically evaluate adjacent mediastinal structures [5]. It is typically reliable for identifying endotracheal nodules and eccentric tracheal wall thickening with tumor growth [2]. Tracheal lesions that are small or confined to the submucosa, however, may present a particular diagnostic challenge, as they can evade detection on routine imaging and may be missed by superficial sampling [6].

In this case, the patient presented with a nonspecific globus sensation, ball-valving sensation, and the ability to generate an audible noise with rapid exhalation. He underwent a chest CT without contrast that revealed tracheal lesions but did not identify the left mainstem bronchus lesion. Although CT is generally sensitive for identifying intraluminal airway lesions, its sensitivity is reduced for small, flat, or submucosal lesions, particularly when contrast is not used [6]. Endoscopic evaluation in this case allowed for direct visualization, revealing additional lesions that were otherwise occult on imaging. This emphasizes the critical role of direct laryngoscopy and bronchoscopy in the workup of patients with persistent airway symptoms and a prior history of malignancy, even if initial imaging appears reassuring.

## Conclusions

Tracheal metastasis from colorectal adenocarcinoma is rare, but clinically significant. Lesions that are very small or in a submucosal location may be missed on routine imaging, delaying diagnosis. Endoscopic examination is an essential component of confirming tracheobronchial lesions. Clinicians should maintain a high level of suspicion and consider direct visualization of the airway in patients with persistent or unexplained symptoms and a known history of malignancy, regardless of initial imaging results. Heightened awareness of this rare metastatic pattern, in addition to early and direct visualization of the airway, may improve diagnostic accuracy and guide patient management.

## References

[1] Wang J, Li S, Liu Y, Zhang C, Li H, Lai B (2020). Metastatic patterns and survival outcomes in patients with stage IV colon cancer: a population-based analysis. Cancer Med.

[2] Choi IY, Lee KY, Lee JH (2013). Tracheal metastasis from rectal cancer: a case report and review of the literature. Balkan Med J.

[3] Serraj M, Lakranbi M, Ghalimi J, Ouadnouni Y, Tizniti S, Smahi M (2013). About a submucosal tracheal tumor. World J Surg Oncol.

[4] Amin MB, Greene FL, Edge SB (2017). The Eighth Edition AJCC Cancer Staging Manual: continuing to build a bridge from a population-based to a more "personalized" approach to cancer staging. CA Cancer J Clin.

[5] Shepard JO, Flores EJ, Abbott GF (2018). Imaging of the trachea. Ann Cardiothorac Surg.

[6] Stevic R, Milenkovic B (2016). Tracheobronchial tumors. J Thorac Dis.

